# Downregulation of miRNA‐126‐3p is associated with progression of and poor prognosis for lung squamous cell carcinoma

**DOI:** 10.1002/2211-5463.12920

**Published:** 2020-07-14

**Authors:** Shang‐Wei Chen, Hui‐Ping Lu, Gang Chen, Jie Yang, Wan‐Ying Huang, Xiang‐Ming Wang, Shu‐Ping Huang, Li Gao, Jun Liu, Zong‐Wang Fu, Peng Chen, Gao‐Qiang Zhai, Jiao Luo, Xiao‐Jiao Li, Zhi‐Guang Huang, Zu‐Yun Li, Ting‐Qing Gan, Da‐Ping Yang, Wei‐Jia Mo, Hua‐Fu Zhou

**Affiliations:** ^1^ Department of Thoracic and Cardiovascular Diseases First Affiliated Hospital of Guangxi Medical University Nanning China; ^2^ Department of Pathology First Affiliated Hospital of Guangxi Medical University Nanning China; ^3^ Department of Pharmacology School of Pharmacy Guangxi Medical University Nanning China; ^4^ Department of Medical Oncology The Second Affiliated Hospital of Guangxi Medical University Nanning China; ^5^ Department of PET/CT First Affiliated Hospital of Guangxi Medical University Nanning China; ^6^ Department of Pathology Guigang People's Hospital of Guangxi/the Eighth Affiliated Hospital of Guangxi Medical University Guigang China

**Keywords:** lung squamous cell carcinoma, MiRNA‐126‐3p, RT‐qPCR, target genes, TCGA

## Abstract

Lung squamous cell carcinoma (LUSC) is the main pathological type of pulmonary malignant tumors; at present, less than 10% of patients with advanced metastatic LUSC live for more than 5 years. We previously reported that low expression of miRNA‐126‐3p is associated with the occurrence and progression of lung adenocarcinoma (LUAD). Here, we examined expression of miRNA‐126‐3p in 23 samples from patients with LUSCs and 23 normal control specimens by quantitative real‐time PCR (RT‐qPCR). Associations between miRNA‐126‐3p expression and clinical features were studied from materials derived from Gene Expression Omnibus (GEO) chips and The Cancer Genome Atlas (TCGA) database. Twelve online platforms were used to identify candidate target genes of miRNA‐126‐3p. Further analyses of the Kyoto Encyclopedia of Genes and Genomes (KEGG), Gene Ontology (GO), and protein–protein interaction (PPI) network were performed on the target genes. GEO microarray analysis, TCGA data mining, RT‐qPCR, and integration analysis consistently reported low expression of miRNA‐126‐3p in LUSC. A total of 42 genes were identified as potential target genes of miRNA‐126‐3p from online platforms, GEO microarrays, and the TCGA database. GO and KEGG analyses demonstrated that the target genes are involved in several biological processes that promote the progression of LUSC. *SOX2*, *E2F2*, and *E2F3* were selected as hub genes from the PPI network for further analysis. In summary, our results suggest that the low expression of miRNA‐126‐3p may play a role in promoting the development of LUSC and miRNA‐126‐3p may be a biomarker for LUSC early diagnosis and prognosis.

## Graphical abstract

Here, we show that miRNA‐126‐3p is significantly underexpressed in 685 cases of lung squamous cell carcinoma (LUSC) as compared to 298 nontumor lung tissues, by combining various methods including in‐house RT‐qPCR, miRNA‐sequencing, and miRNA microarrays. The downregulation of miRNA‐126‐3p may regulate cell cycle signaling by targeting *E2F2* and *E2F3*, and this may result in LUSC carcinogenesis.

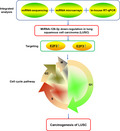

**Abbreviations**
AUC: area under the curve
BP: biological process
CC: cellular component
CI: confidence interval
DAVID: Database for Annotation, Visualization and Integrated Discovery
DEGs: differentially expressed genes
FFPE: formalin‐fixed, paraffin‐embedded
GEO: Gene Expression Omnibus
GO: Gene Ontology
KEGG: Kyoto Encyclopedia of Genes and Genomes
LR: likelihood ratio
LUAD: lung adenocarcinoma
LUSC: lung squamous cell carcinoma
MF: molecular function
NSCLC: non‐small‐cell lung carcinoma
OR: odds ratio
OS: overall survival
PPI: protein–protein interaction
Q: quartile
ROC: receiver operating characteristic
RT‐qPCR: quantitative real‐time PCR
SCLC: small cell lung cancer
SD: standard deviation
SE: standard error
SMD: standard mean difference
SROC: summary receiver operating characteristic
STRING: Search Tool for the Retrieval of Interacting Genes
TCGA: The Cancer Genome Atlas
TFs: transcription factors

Lung cancer is a neoplastic disease of the lower respiratory tract; it includes a variety of histological subtypes and biologically distinct genotypes that have a high mortality rate [1]. The annual incidence of lung cancer worldwide is about 1.8 million, and it is the second most common cancer among both males and females, accounting for about 14% of all cancers [1]. Lung squamous cell carcinoma (LUSC) is the main pathological type of pulmonary malignant tumors; unlike pulmonary adenocarcinoma, it is more common in men and smokers [2, 3]. In people with a smoking history of at least 30 years, the risk index for LUSC is 5.5 times that of nonsmokers [4]. Similar to cervical cancer, LUSC is thought to progress through the process of dysplasia, and it develops into an aggressive cancer within a few years [5]. At present, the overall prognosis of patients with advanced LUSC is unsatisfactory, and less than 10% of patients with advanced metastatic LUSC live for more than 5 years [6, 7, 8]. To improve prognosis, we must first determine the underlying molecular mechanisms.

MicroRNAs (miRNA) have a variety of pathophysiological functions and regulate gene expression through translational inhibition and inducing mRNA instability [9, 10]. Recent studies indicate that many miRNAs are associated with LUSC; for example, Qi *et al*. [11] found that seven highly expressed miRNAs (miRNA‐301b, miRNA‐383, miRNA‐512, miRNA‐515, miRNA‐525, miRNA577, and miRNA5682) and five underexpressed miRNAs (miRNA‐448, miRNA‐486, miRNA‐4732, miRNA‐516, and miRNA‐1911) are associated with improved prognosis of patients with LUSC. In previous studies, we reported that miRNA‐136‐5p [12], miRNA‐182‐5p [13], miRNA‐198‐5p [14], miRNA‐452‐5p [15], and miRNA‐375 [16] are all correlated with the development and prognosis of LUSC. MiRNA‐126‐3p is located on human chromosome 9, and it is associated with thyroid cancer [17] and pancreatic cancer [18]. Our previous findings indicate that low expression of miRNA‐126‐3p is associated with the occurrence and progression of lung adenocarcinoma (LUAD) [19]. However, there are few reports on the specific mechanism of the role of miRNA‐126‐3p in LUSC. Here, we aimed to elucidate the specific molecular action of miRNA‐126‐3p in LUSC to help provide a new direction for the early detection and treatment of LUSC.

In this study, the expression pattern and clinical role of miRNA‐126‐3p in LUSC were determined by quantitative real‐time PCR (RT‐qPCR). The association between the expression of miRNA‐126‐3p and clinical features was studied using materials derived from Gene Expression Omnibus (GEO) chips, related literature, and the Cancer Genome Atlas (TCGA). Subsequently, 12 online analyses were performed to identify candidate target genes of miRNA‐126‐3p, and the potential mechanism of miRNA‐126‐3p in LUSC was studied by examination of key genes, such as *SOX2*, *E2F2*, and *E2F3*.

## Materials and methods

### Clinical samples

All clinical samples collected were derived from LUSC patients who underwent radical surgery for lung cancer at the First Affiliated Hospital of Guangxi Medical University. The samples were routinely made into paraffin blocks, and 5‐µm‐thick slides were prepared for the pathological observation. All the LUSC samples were screened, and only those with a tumor cell ratio greater than 75% were included in the current study. Ultimately, 23 formalin‐fixed, paraffin‐embedded (FFPE) LUSC tissues in total and corresponding adjacent noncancerous lung tissues were gathered from the Department of Pathology of the First Affiliated Hospital of Guangxi Medical University (Nanning, Guangxi, China) from January 2012 to February 2014. Two pathologists (Zu‐Yun Li and Gang Chen) confirmed the diagnosis and tumor cell content separately. The demographic and clinical data of the 23 patients are presented in Table 1. The Ethics Committee of the First Affiliated Hospital of Guangxi Medical University authorized the research protocol (approval number: 2015‐KY‐NSFC‐059), and written informed consent was obtained from each participant. The study complied with the Declaration of Helsinki, as well as with applicable local laws and regulations.

**Table 1:** Clinical parameters of 23 LUSC patients.

Clinicopathological feature	Category	*N*	%
Tissue	LUSC	23	–
	Noncancerous tissue	23	–
Age (years)	< 60	15	65.2
	≥ 60	8	34.8
Gender	Female	5	21.7
	Male	18	78.3
Tumor size (cm)	≤ 3	7	30.4
	> 3	16	69.6
Smoke state	No	12	52.1
	Yes	11	47.9
T stage	T1–2	15	65.2
	T3–4	8	34.8
Lymph node metastasis	No	11	47.9
	Yes	12	52.1
Vascular invasion	No	20	86.9
	Yes	3	13.1
TNM stage	I–II	10	43.4
	III–IV	13	56.6
Pathological grading	II	16	69.5
	III	7	30.5

### Quantitative real‐time PCR

The thickness of the clinical FFPE tissue section was 10 μm for the RNA extraction in the RT‐qPCR procedure. Three sections were used to isolate the total RNA, which was later proven to yield an optimal RNA quantity and quality. Xylene and ethanol were utilized to de‐wax the tissues. In accordance with the manufacturer's procedure, total RNA was isolated from tumor sections using the miRNeasy FFPE Kit (Qiagen, KJ Venlo, the Netherlands) including a genomic DNA (gDNA) elimination step. We prolonged the incubation time with protease K to 36 h at 55 °C. Next, protease K was added every 12 h to maintain its concentration. Contaminating DNA was eliminated from RNA extract by using RNase‐free DNase (Qiagen) based on the manufacturer's recommendations. According to the size of the tumor sample, the RNA concentration ranged from 20 to 2 μg·μL^−1^ and the quality of the isolated RNA was detected by NanoDrop 2000 UV spectrophotometer (Wilmington, DE, USA). RNA purity was evaluated by calculating the 260/280 and 260/230 absorbance ratios. The 260/280 values of 1.8–2.0 and 260/230 values in the range of 1.8–2.2 demonstrate the RNA was free of contamination. Lower ratios may indicate the presence of protein, peptides, aromatic compounds, phenol, carbohydrates, or other contaminants that absorb at or near 260, 230, or 280 nm. RNA integrity was measured using an RNA Nano 6000 Assay Kit and a Bioanalyzer 2100 system (Agilent Technologies, Santa Clara, CA, USA) and assessed according to the RNA Integrity Number (RIN) that classified total RNA on a numbering system from 1 (the most degraded) to 10 (the most intact). Then, reverse transcription synthesis of complimentary DNA (cDNA) was conducted on First Strand cDNA Synthesis Kit (Thermo Scientific, Waltham, MA, USA) with adding 100 ng total RNA to a final volume of 10 μL, followed by PCR reaction on an Applied Biosystems PCR7900 instrument (Thermo Fisher Scientific, Waltham, MA, USA). The specific primer of miRNA‐126‐3p was provided by TaqMan MicroRNA Assays (4427975‐000468; Applied Biosystems, Life Technologies Europe B.V, Bleiswijk, Netherlands). The reverse primers were applied in the reverse transcription step with TaqMan MicroRNA Reverse Transcription Kit (4366596; Applied Biosystems, Life Technologies Europe B.V) in a total volume of 10 μL. The thermocycling steps were as follows: denaturation at 95 °C for 10 min, followed by 40 cycles of 95 °C for 15 s and 60 °C for 1 min. We executed RT‐qPCR on the 7900HT PCR system (Applied Biosystems) [20, 21, 22]. RNU6B was considered a stable endogenous control. The sequences of RNU6B and miRNA‐126‐3p were CGCAAGGAUGACACGCAAAUUCGUGAAGCGUUCCAUAUUUUU and UCGUACCGUGAGUAAUAAUGCG, respectively. The formula for 2^−Δcq^ was utilized to calculate the expression value of miRNA‐126‐3p.

### Extraction of miRNA‐126‐3p expression from TCGA

We downloaded and extracted the miRNA‐126‐3p expression profile and related clinical features in LUSC from TCGA (https://tcga‐data.nci.nih.gov/docs/publications/tcga/). We standardized the extracted data and converted to log2. Then, ibm spss statistics V23.0 software (IBM Corp., Armonk, NY, USA) was applied to analyze statistically the expression level of miRNA‐126‐3p and the correlation with the clinical data. We used the intermediate expression level of miRNA‐126‐3p to construct a Kaplan–Meier curve to describe the relationship between the aberrant expression of miRNA‐126‐3p and the overall survival (OS) rate of LUSC patients. To analyze the prognostic factors of LUSC, the r‐software (Lucent Technologies, Jasmine Hill, NJ, USA) function package was used to perform univariate and multivariate Cox analyses of the clinically relevant traits of LUSC.

### Collection of miRNA‐126‐3p related datasets

We excavated chips from the databases and literatures of the GEO (https://www.ncbi.nim.nih.gov/geo/), ArrayExpress (https://www.ebi.ac.uk/arrayexpress/), SRA (https://www.sra.org.uk/), and Oncomine (https://www.oncomine.org/). The search terms and methods we used to screen the database chips and related literature were the same as those used in previous research [13, 14, 15, 23]. We used ‘Homo sapiens [Organism]’ to limit the search. The selection criteria for microarrays were as follows: (a) The microarray involved LUSC and adjacent tissue specimens, and (b) the study provided data on the miRNA‐126‐3p original expression profile in LUSC and noncancerous tissues. According to the following exclusion criteria, the microarray was considered unqualified if (a) the microarray was from cell line, (b) there was a small sample size in the LUSC group or noncancer group (*n* < 3), and (c) the study included only LUSC samples and no controls [13].

### Integrated analysis of miRNA‐126‐3p expression by multiple methods

We integrated the RT‐qPCR results, TCGA data, and GEO dataset in the stata 14.0 software (Stata Corp LP, College Station, TX, USA) to assess the miRNA‐126‐3p expression pattern in LUSC. The standard mean difference (SMD) and 95% confidence interval (CI) were calculated to evaluate the miR‐126‐3p expression in LUSC and normal samples. Specifically, if the heterogeneity was low (*I* ^2^ ≤ 50% and *P* ≥ 0.05), the fixed effect model could be chosen; otherwise, we used the random effect model [24, 25]. The summary receiver operating characteristic (SROC) curve was constructed based on the integrated data to evaluate the potential discrimination ability of miRNA‐126‐3p in LUSC.

### Prediction and confirmation of miRNA‐126‐3p target genes

According to the method our team used previously, we used 12 online databases (miRWalk, Microt4, miRanda, mirbridge, miRDB, miRMap, miRNAMap, Pictar2, PITA, RNAhybrid, Targetscan, and RNA22) (http://zmf.umm.uni‑heidelberg.de/apps/zmf/mirwalk2/miRretsys‑self.html) to predict the target genes of miRNA‐126‐3p [12, 13, 14, 15, 23]. The genes involved in at least three predictive programs were considered candidate target genes for miRNA‐126‐3p. In light of the relationship between miRNA and mRNA, a decreased expression of miRNA may result in an overexpression of the target gene, so we screened the overexpressed gene in the TCGA and GEO databases as the candidate target gene for prediction and filtered all overexpressed genes in LUSC from the TCGA with the limma package of r. The criteria were logFC > 1 and adj.*P* < 0.05. We used the same method and standard to screen the overexpressed genes in 20 LUSC‐related microarrays obtained from the GEO database, namely GSE6044, GSE11117, GSE11969, GSE12472, GSE19188, GSE21933, GSE27489, GSE29927, GSE30118, GSE30219, GSE31446, GSE31552, GSE33479, GSE33532, GSE40275, GSE51852, GSE67061, GSE68606, GSE84784, and GSE103512. Finally, the target genes of miRNA‐126‐3p were obtained by overlapping the candidate target genes predicted online with the overexpressed genes obtained from TCGA and GEO databases. Further, KEGG, GO, and PPI network analyses were performed on the target genes.

### Functional analysis for promising target genes

The Database for Annotation, Visualization, and Integrated Discovery (DAVID) (https://david.ncifcrf.gov/) was applied to the GO and KEGG analyses. The GO and KEGG enrichment analyses were visualized using the r (Lucent Technologies) function ggplot2. A *P* value of < 0.05 was selected, as valid items enriched in target genes. A PPI network was established using the Search Tool for the Retrieval of Interacting Genes (STRING) v.11.0 (http://www.string‐db.org/).

### Statistical analysis

The ibm spss statistics V23.0 software (IBM Corp.) was used to undertake the statistical analysis. The calculated results were presented in the form of the mean ± standard deviation (SD). The Mann–Whitney *U*‐test or Student's *t*‐test was executed to evaluate the difference between the two variables. The receiver operating characteristic (ROC) curve was drawn, and the area under the curve (AUC) was calculated to judge the discrimination power of miRNA‐126‐3p in LUSC. Statistical changes were considered to occur when *P* < 0.05.

## Results

### Validation of the expression of miRNA‑126‐3p using RT‑qPCR

**Fig. 1:**
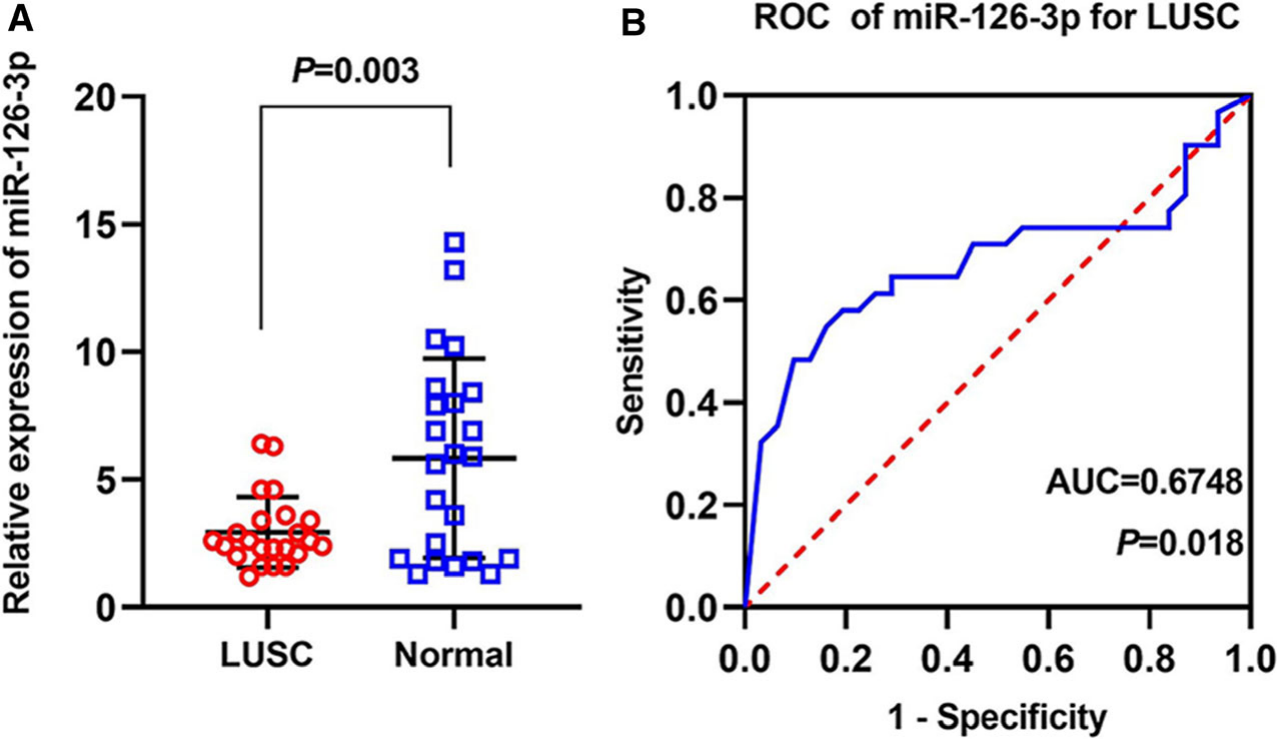
Diagnostic value of miRNA‐126‐3p for LUSC according to RT‐qPCR. (A) The expression of miRNA‐126‐3p in 23 LUSC and 23 normal tissues. (B) The ROC curve was generated to assess the diagnostic ability of miRNA‐126‐3p in 23 LUSC and 23 normal tissues. The AUC 95% CI: 0.5358–0.8161. AUC: 0.5–0.7 (low), 0.7–0.9 (moderate), and 0.9–1.0 (high). Data are expressed as means ± SD, and *P* < 0.05 indicated statistically significant difference when compared to the normal control. Comparisons among two groups were carried out with Student's paired *t*‐test.

The 260/280 ratios were detected, and RNA concentrations ranged from 20 to 2 μg·μL^−1^. The ranges of the 260/280 and 260/230 ratios were 1.85–2.05 and 1.89–2.03, respectively. The amplification efficiency of PCR in our study was 90.2–95.1%. According to geNorm and NormFinder, the gene which has the smallest stability value is the candidate gene most stably expressed in the sample set investigated. Therefore, the housekeeping gene RNU6B was used as an internal reference in the RT‐qPCR as evidenced by our previous work with NormFinder and geNorm (Fig. S1 and Table S1). We examined the clinicopathological value of miRNA‐126‐3p in 23 LUSCs and 23 normal control specimens by RT‐qPCR and found that the miRNA‐126‐3p expression level in LUSC was significantly lower than that of normal tissues (2.943 ± 1.378 vs 5.841 ± 3.897, *P = *0.003, Fig. 1A and Table 2). The ROC curve of miRNA‐126‐3p in the LUSC tissue demonstrated that the AUC was 0.6748 (*P = *0.018; Fig. 1B). No differences were found in other related clinicopathological features of miRNA‐126‐3p (all *P* > 0.05, Table 2).

**Table 2:** Associations between miRNA‐126‐3p expression and clinicopathological features in LUSC based on in‐house RT‐qPCR data and TCGA miRNA‐seq data.

Data sources	Clinicopathological feature	Category	*N*	mean ± SD	*P* value
In‐house RT‐qPCR	Tissue	LUSC	23	2.94 ± 1.37	0.003*
		Noncancerous	23	5.84 ± 3.89	
	Age (years)	< 60	15	2.70 ± 1.32	0.164
		≥ 60	8	3.38 ± 1.45	
	Gender	Female	5	2.88 ± 2.02	0.549
		Male	18	2.96 ± 1.22	
	Tumor size (cm)	≤ 3	7	2.77 ± 0.98	0.814
		> 3	16	3.01 ± 1.54	
	Smoke	No	12	3.20 ± 1.70	0.516
		Yes	11	2.66 ± 0.90	
	T stage	T1–2	15	3.30 ± 1.50	0.089
		T3–4	8	2.27 ± 0.82	
	Lymph node metastasis	No	11	3.35 ± 1.76	0.196
		Yes	12	2.56 ± 0.81	
	Vascular invasion	No	20	3.02 ± 1.43	0.492
		Yes	3	2.43 ± 0.90	
	TNM stage	I–II	10	3.47 ± 1.73	0.147
		III–IV	13	2.53 ± 0.90	
	Pathological grading	II	16	2.78 ± 0.94	0.920
		III	7	3.31 ± 2.12	
TCGA miRNA‐seq	Tissue	LUSC	478	11.44 ± 1.00	< 0.001*
		Noncancerous	45	12.65 ± 0.80	
	Age (years)	< 60	87	11.45 ± 0.94	0.954
		≥ 60	383	11.43 ± 1.02	
	Gender	Female	124	11.55 ± 0.96	0.110
		Male	354	11.40 ± 1.01	
	Pathological stage	I–II	388	11.44 ± 1.02	0.705
		III–IV	88	11.47 ± 0.91	
	T stage	T1–T2	387	11.48 ± 0.98	0.021*
		T3–T4	91	11.25 ± 1.05	
	Node	No	306	11.48 ± 1.02	0.194
		Yes	172	11.36 ± 0.96	
	Metastasis	No	472	11.44 ± 1.00	0.527
		Yes	5	11.20 ± 0.67	
	Anatomic subdivision	L_lower	70	11.51 ± 0.93	0.783^a^
		L_upper	128	11.38 ± 0.99	
		R_lower	106	11.55 ± 1.05	
		R_middle	15	11.59 ± 1.26	
		R_upper	131	11.43 ± 0.98	
		Bronchial	10	11.29 ± 1.01	
	Tumor location	Peripheral	91	11.32 ± 0.97	0.775
		Central	140	11.28 ± 1.02	

^a^ Kruskal–Wallis test was performed to assess the distribution difference of miR‐126‐3p in three or more groups of clinicopathological parameters.

* *P* < 0.05 was considered statistically significant. Comparisons among two groups were carried out with Student's paired *t*‐test in RT‐qPCR and unpaired two‐sided Mann–Whitney *U*‐test in TCGA.

### Confirmation the relationships between miRNA‐126‐3p and clinical pathology of LUSC, according to TCGA

**Fig. 2:**
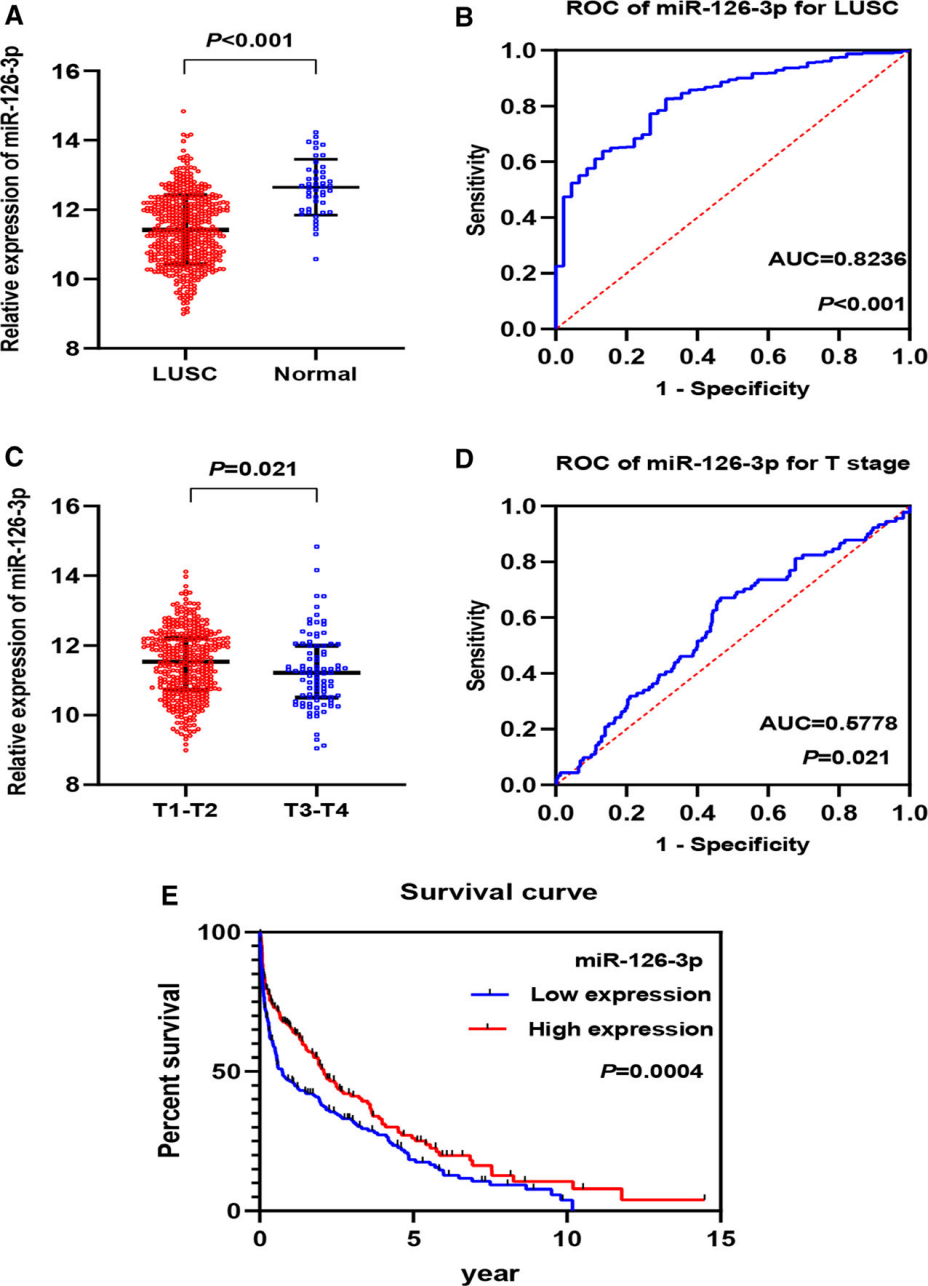
Relationship between miRNA‐126‐3p expression and T stage in LUSC and diagnostic value of miRNA‐126‐3p expression according to TCGA. (A) The expression of miRNA‐126‐3p in 478 LUSC and 45 noncancerous lung tissues. (B) The ROC curve was generated to assess the diagnostic ability of miRNA‐126‐3p in 478 LUSC and 45 noncancerous lung tissues. The AUC 95% CI: 0.7743–0.8829. (C) Expression of miRNA‐126‐3p in early (T1–T2) and late (T3–T4) T stages of LUSC. (D) ROC curve of miRNA‐126‐3p for T stages of LUSC (95% CI: 0.5130–0.6426). (E) The Kaplan–Meier curve of LUSC patients. AUC: 0.5–0.7 (low), 0.7–0.9 (moderate), and 0.9–1.0 (high). Data are expressed as means ± SD, and *P* < 0.05 indicated statistically significant difference when compared to the normal control. Comparisons among two groups were carried out with unpaired two‐sided Mann–Whitney *U*‐test.

**Fig. 3:**
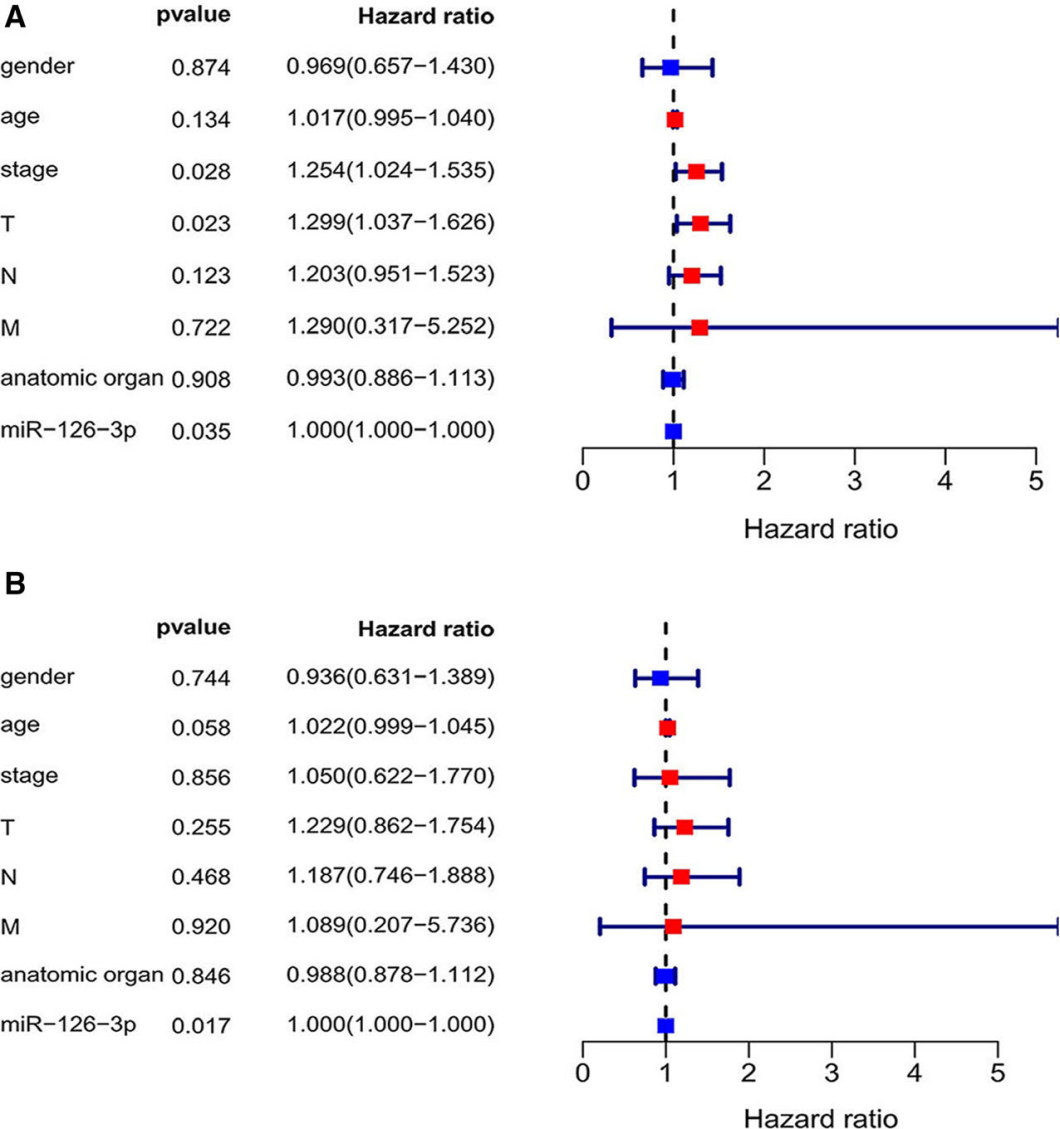
Cox regression analysis for the prognostic value of miRNA‐126‐3p and LUSC clinicopathological parameters according to TCGA. (A) Univariate Cox regression analysis. (B) Multivariate Cox regression analysis. *P* < 0.05 indicated statistically significant difference.

In total, 478 tumor tissues and 45 noncancerous tissues were obtained for the expression data and clinical information of miRNA‐126‐3p from TCGA. Compared with the noncancerous group (12.65 ± 0.802), the miRNA‐126‐3p expression was greatly decreased in the LUSC group (11.44 ± 1.003) (*P* < 0.001; Table 2; Fig. 2A). The ROC curve was utilized to assess the recognition capability of miRNA‐126‐3p in LUSC (AUC = 0.8236, *P* < 0.001; Fig. 2B). The miRNA‐126‐3p expression differed remarkably in the T stage; it was higher in the T1–T2 stage than in the T3–T4 stage (*P* = 0.021; Fig. 2C). As shown in Fig. 2D, the AUC of the T stage was 0.5778 (*P* = 0.021). No difference was found between the other clinicopathological features of miRNA‐126‐3p (all *P* > 0.05, Table 2). Figure 2E illustrates the relationship between miRNA‐126‐3p expression levels and LUSC survival. It was clear that LUSC patients with a high expression of miRNA‐126‐3p had significantly better OS than patients with a low expression (*P* = 0.0004). The relative expression and clinicopathological features of miRNA‐126‐3p were analyzed by univariate and multivariate Cox analyses, and miRNA‐126‐3p was found to be significantly different in the univariate and multivariate Cox analyses compared with other clinicopathological characteristics (Fig. 3A,B). These results indicate that miRNA‐126‐3p might act as a tumor inhibitor in LUSC, and it might suppress the proliferation of LUSC cells.

### GEO data mining for miRNA‐126‐3p

**Fig. 4:**
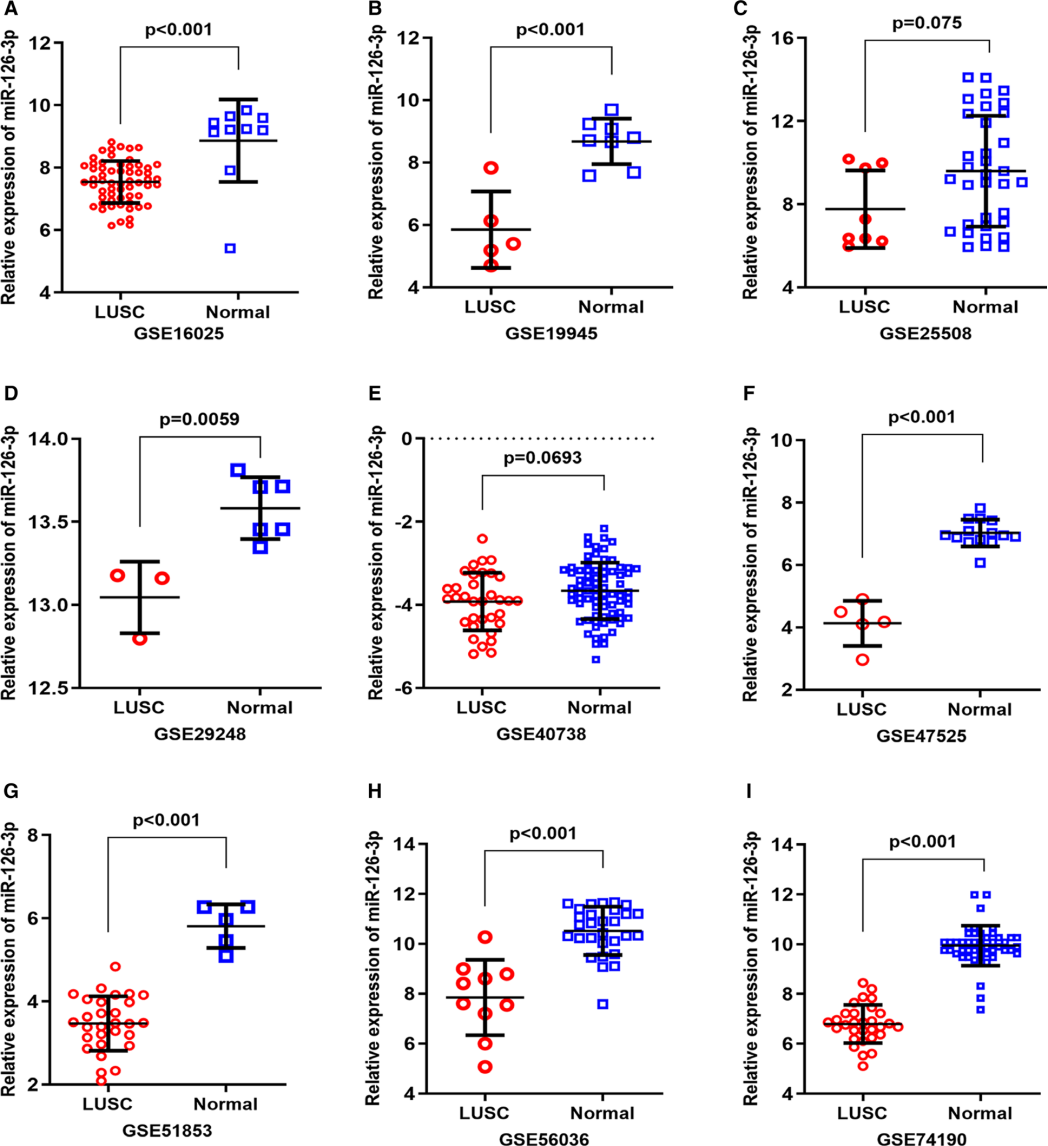
MiRNA‐126‐3p expression in LUSC tissues according to GEO microarrays. The scatter plots display the differential expression levels of miRNA‐126‐3p in LUSC and noncancer tissues for each of the included GSE datasets. (A) GSE16025. (B) GSE19945. (C) GSE25508. (D) GSE29248. (E) GSE40738. (F) GSE47525. (G) GSE51853. (H) GSE56036. (I) GSE74190. Data are expressed as means ± SD, and *P* < 0.05 indicated statistically significant difference when compared to the normal control. Comparisons among two groups were carried out with unpaired two‐sided Mann–Whitney *U*‐test.

**Fig. 5:**
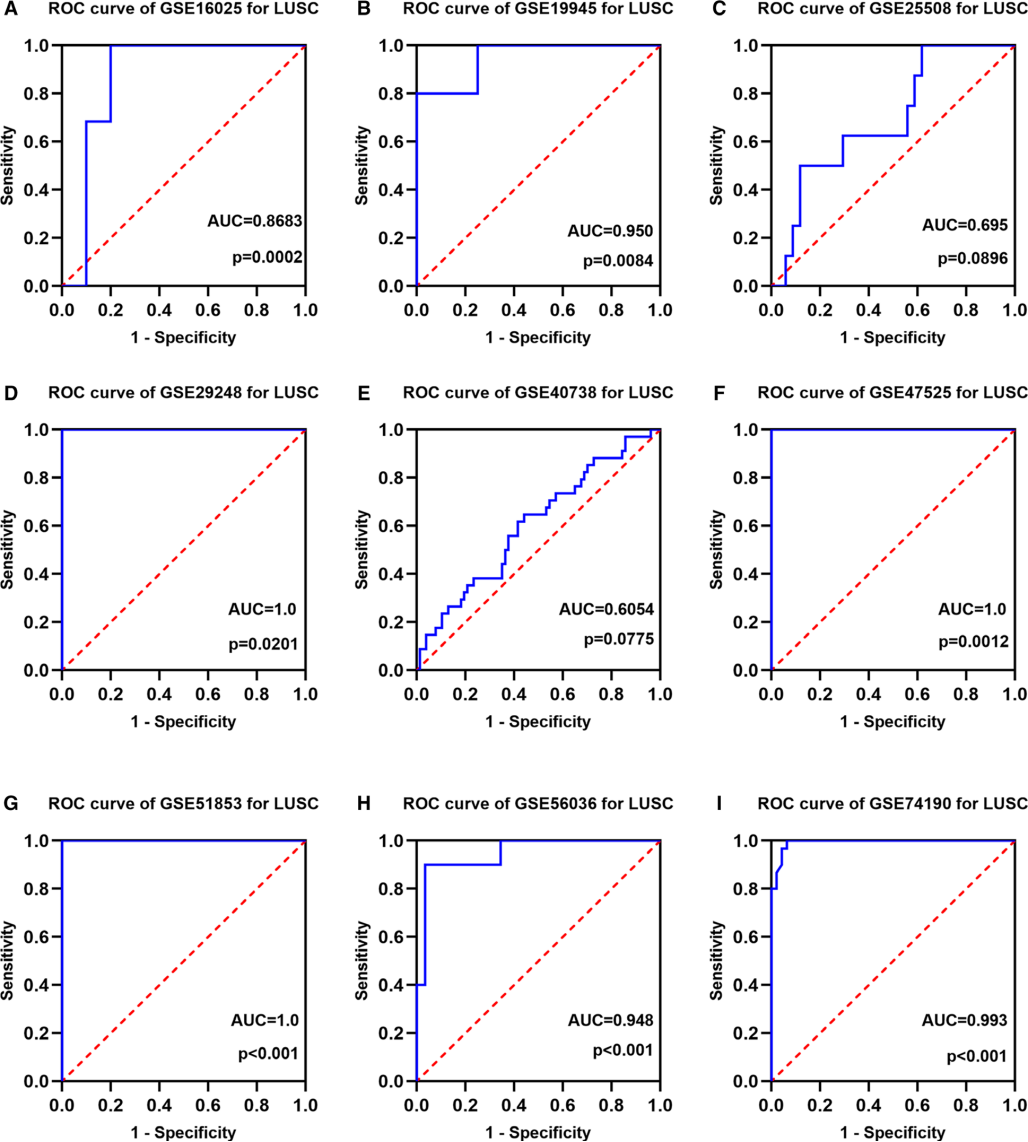
ROC curves based on GSE datasets. A panel of ROC curves shows the diagnostic ability of miRNA‐126‐3p for LUSC in each of the included GSE datasets. (A) GSE16025. (B) GSE19945. (C) GSE25508. (D) GSE29248. (E) GSE40738. (F) GSE47525. (G) GSE51853. (H) GSE56036. (I) GSE74190. AUC: 0.5–0.7 (low), 0.7–0.9 (moderate), and 0.9–1.0 (high). *P* < 0.05 indicated statistically significant difference.

We screened nine microarrays from the GEO dataset, namely GSE29248, GSE40738, GSE16025, GSE51853, GSE19945, GSE25508, GSE47525, GSE56036, and GSE74190. A scatter plot was used to illustrate the content of miRNA‐126‐3p in LUSC and normal tissue for each GSE dataset (Fig. 4). The content of miRNA‐126‐3p in LUSC tissues was lower than that of normal tissues in all microarrays. The ROC curve evaluated the diagnostic power of miRNA‐126‐3p among GSE datasets (Fig. 5).

### Comprehensive analysis of TCGA, GEO, and RT‐qPCR

**Fig. 6:**
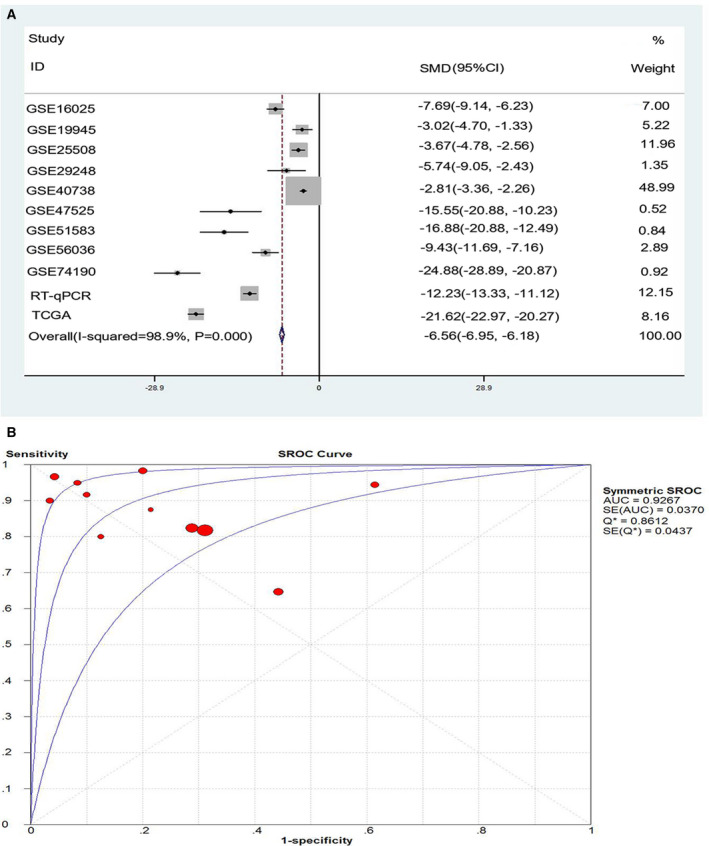
Integrated analysis of RT‐qPCR, TCGA database, and GEO microarrays. (A) Forest plot of miRNA‐126‐3p expression data from RT‐qPCR, TCGA database, and GEO microarrays. (B) SROC curve (AUC) of miRNA‐126‐3p in the diagnosis ability of LUSC data from RT‐qPCR, TCGA, and GEO. *P* < 0.05 indicated statistically significant difference.

**Fig. 7:**
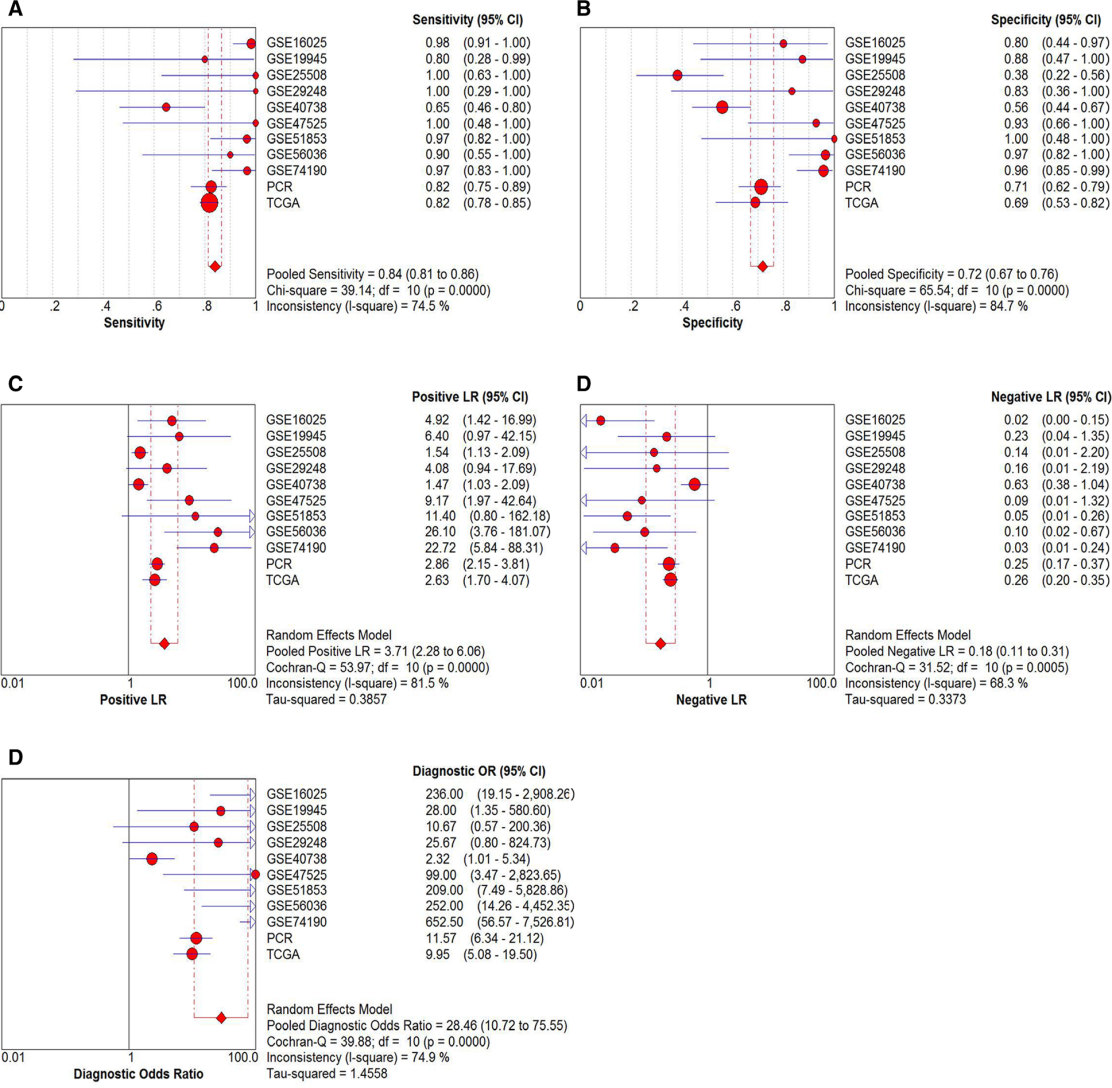
Pooled diagnostic indices for the integrated analysis based on all included studies. (A) The pooled sensitivity for the included studies was 0.84 (0.81–0.86). (B) The pooled specificity was 0.72 (0.67–0.76). (C) The pooled positive LR was 3.71 (2.28–6.06). (D) The pooled negative LR was 0.18 (0.11–0.31). (E) The pooled diagnostic OR was 28.46 (10.72–75.55).

We analyzed the results of TCGA database, GEO chips, and RT‐qPCR integratively. Because the square value of *I* ^2^ was 0.989, a stochastic effect model was used in the analysis. The forest plot showed the expression of miRNA‐126‐3p in each dataset and the results after the integrated analysis (Fig. 6A). According to the random effect model, the combined SMD of miRNA‐126‐3p was −6.56 (95% CI was −6.95 to −6.18), and the *I* ^2^ value was 0.989, *P* < 0.001, which indicated considerable heterogeneity in the study. Figure 6B shows the SROC curve of integrated miRNA‐126‐3p in LUSC with an AUC of 0.9427. We analyzed the integrative sensitivity, specificity, positive likelihood ratio, negative likelihood ratio, and diagnostic ratio of miRNA‐126‐3p in LUSC (Fig. 7). All of these implied that miRNA‐126‐3p showed high diagnostic efficiency in LUSC.

### Prediction of the target genes

**Fig. 8:**
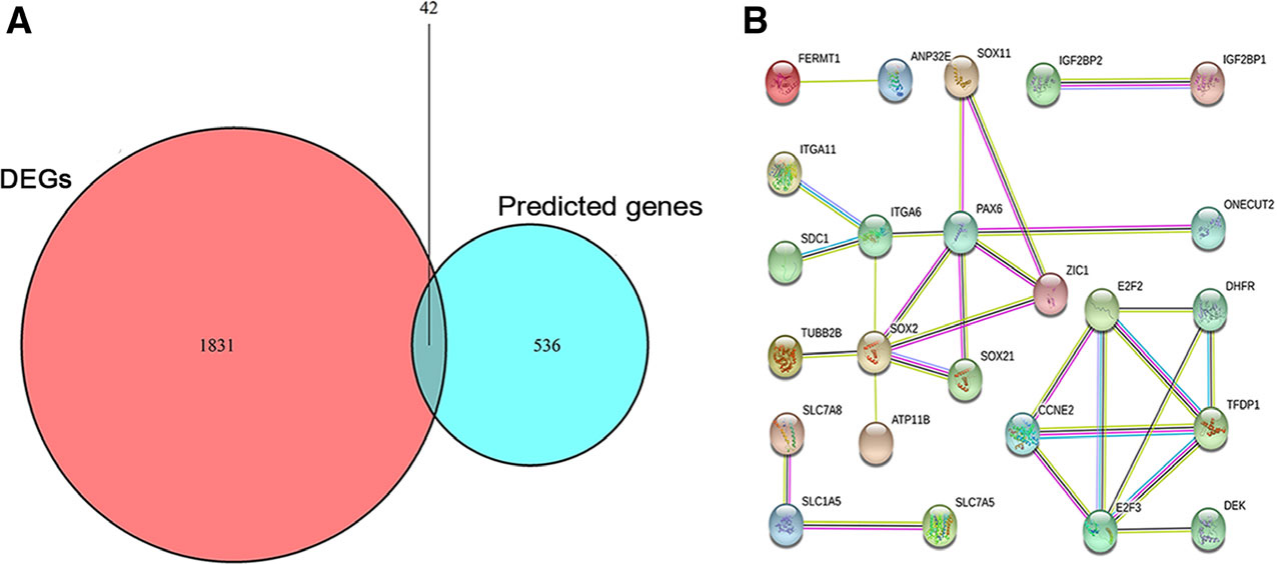
Venn diagram and cluster analysis of the PPI network. (A) Venn diagram of overlapping genes from intersection of two independent datasets. (B) 42 target genes were filtered into the PPI network complex that contained 24 nodes and 28 edges.

Using the 12 online tools mentioned above, we collected 578 candidate target genes in total for miRNA‐126‐3p. In addition, 1873 upregulated genes in LUSC tissues were obtained from TCGA and GEO microarrays. After overlapping these upregulated genes with the 578 candidate targets of miRNA‐126‐3p, 42 genes were obtained as predicted target genes of miRNA‐126‐3p (Fig. 8A).

### Bioinformatics analysis using target genes

**Fig. 9:**
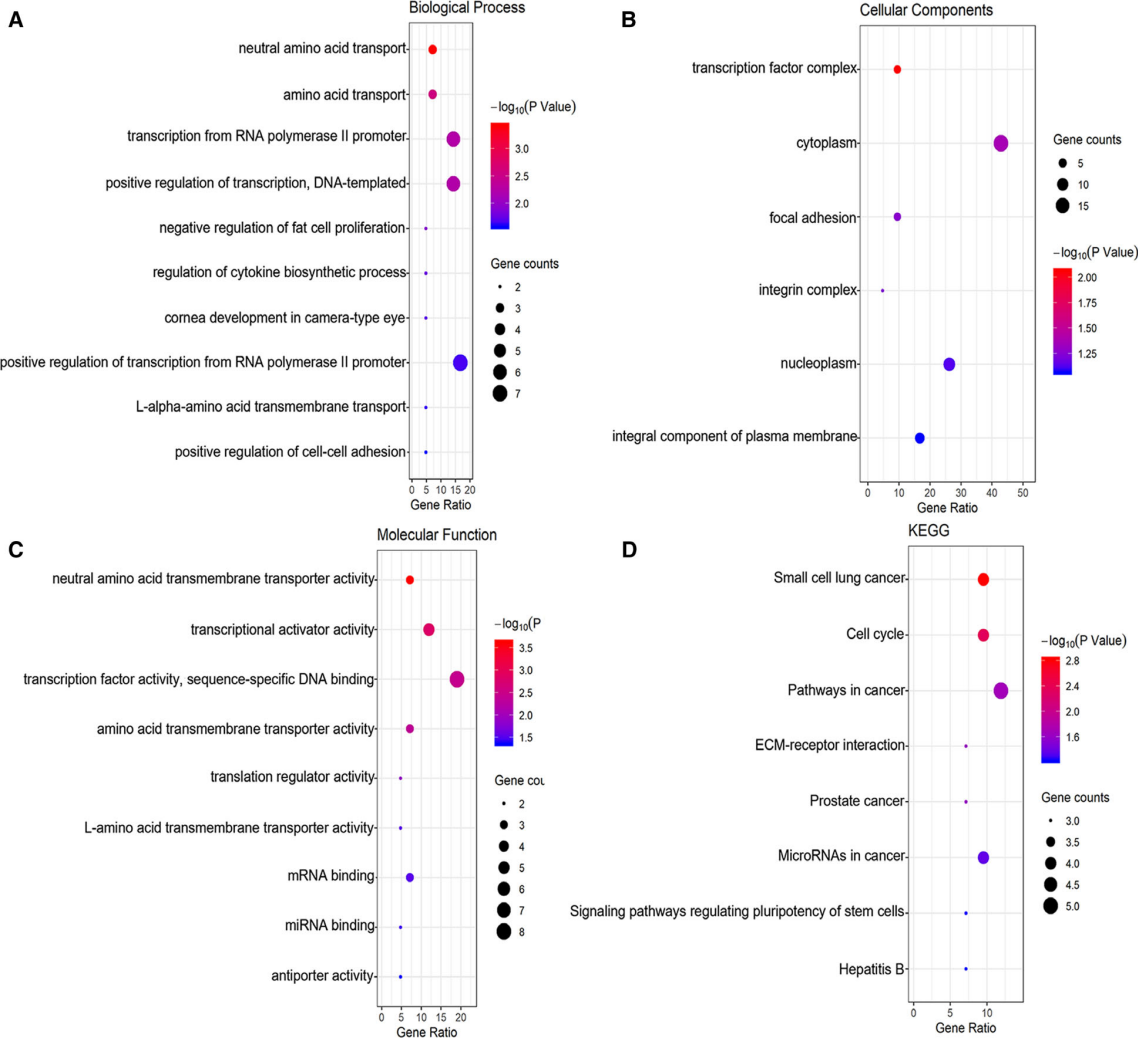
GO analysis and KEGG pathway analysis of the overlapping differentially expressed genes. (A) Top 10 of biological process. (B) Top 6 of cellular component. (C) Top 9 of molecular function. (D) Top 8 of KEGG pathway enrichment.

The predicted 42 target genes were used for GO and KEGG enrichment analyses to explore their potential molecular biological functions concerning LUSC. The GO analysis included three aspects: biological process (BP) (Fig. 9A), cellular component (CC) (Fig. 9B), and molecular function (MF) (Fig. 9C). There was considerable variation in the GO enrichment results for *P* < 0.05. Figure 9D demonstrated significant differences in the KEGG pathways with *P* < 0.05. The most striking KEGG pathway annotations are shown in Table 3. The 42 predicted target genes were also used in the construction of PPI networks. We imported 42 target genes into the STRING network online tool to construct a PPI network with 24 nodes and 28 edges (Fig. 8B). The more connections and occurrences of proteins in the network, the more important the protein was in LUSC. According to the PPI network, *SOX2*, *E2F2*, and *E2F3* are hub genes in LUSC.

**Table 3:** KEGG pathway enrichment analysis of miRNA‐126‐3p.

Term	Count	*P* value	Genes
hsa05222: Small cell lung cancer	4	0.001529	*CCNE2*, *E2F2*, *E2F3*, *ITGA6*
hsa04110: Cell cycle	4	0.004486	*CCNE2*, *E2F2*, *E2F3*, *TFDP1*
hsa05200: Pathways in cancer	5	0.020513	*CCNE2*, *E2F2*, *E2F3*, *ITGA6*, *FZD7*
hsa04512: ECM‐receptor interaction	3	0.023518	*SDC1*, *ITGA6*, *ITGA11*
hsa05215: Prostate cancer	3	0.024026	*CCNE2*, *E2F2*, *E2F3*
hsa05206: MicroRNAs in cancer	4	0.042077	*CCNE2*, *E2F2*, *E2F3*, *IGF2BP1*

### Clinical expression of hub genes

**Fig. 10:**
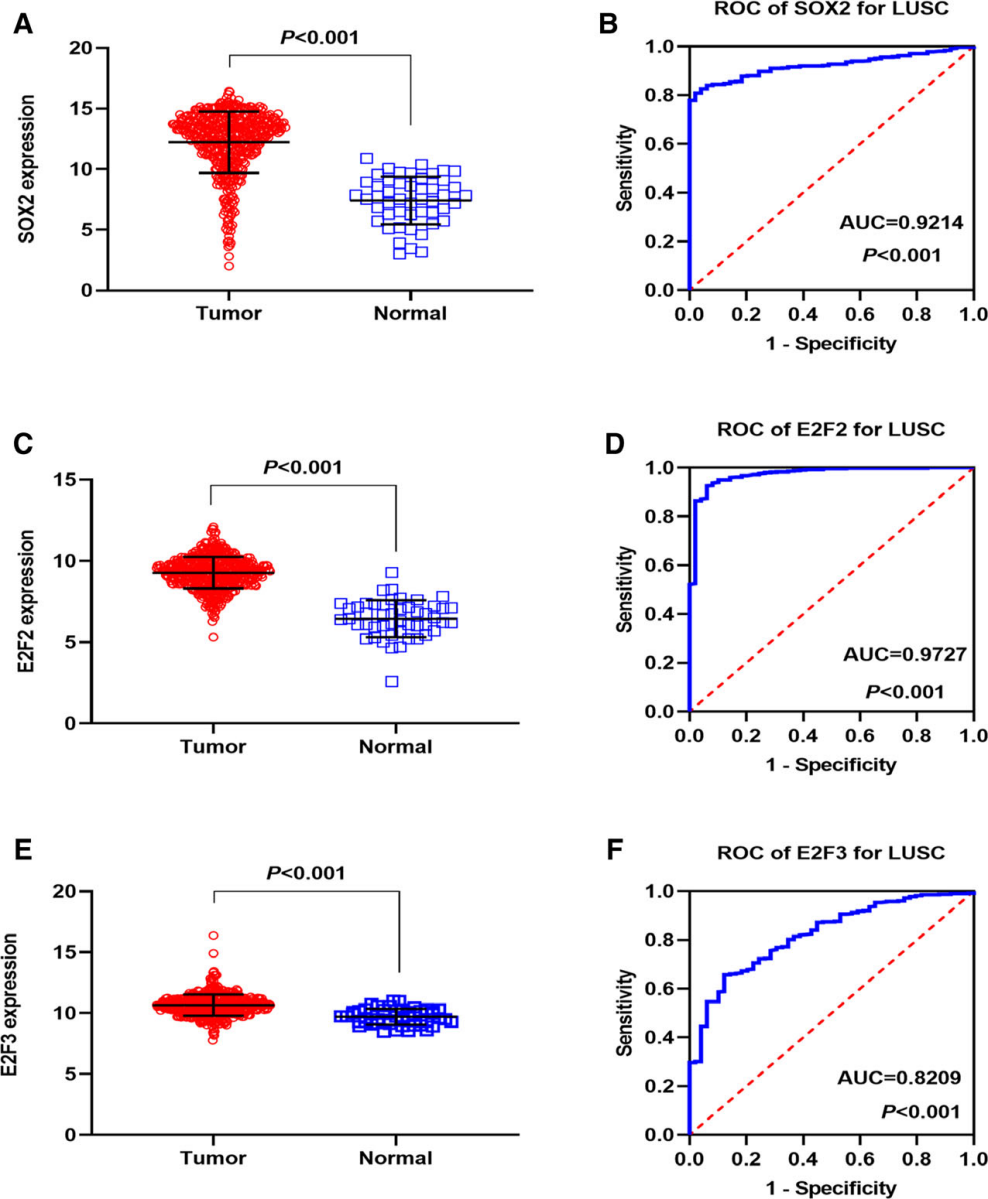
The expression of three hub genes was increased in TCGA LUSC samples and ROC curve analysis. (A) The expression of *SOX2* in 502 LUSC and 49 noncancerous lung tissues. (B) ROC curve was generated to assess the diagnostic ability of *SOX2* in 502 LUSC and 49 noncancerous lung tissues. The AUC was 0.9214 (*P* < 0.001). (C) The expression of *E2F2* in 502 LUSC and 49 noncancerous lung tissues. (D) The ROC curve was generated to assess the diagnostic ability of *E2F2* in 502 LUSC and 49 noncancerous lung tissues. The AUC was 0.9727 (*P* < 0.001). (E) The expression of *E2F3* in 502 LUSC and 49 noncancerous lung tissues. (F) The ROC curve was generated to assess the diagnostic ability of *E2F3* in 502 LUSC and 49 noncancerous lung tissues. The AUC was 0.8209 (*P* < 0.001). Data are expressed as means ± SD, and *P* < 0.05 indicated statistically significant difference when compared to the normal control. Comparisons among two groups were carried out with unpaired two‐sided Mann–Whitney *U*‐test.

**Fig. 11:**
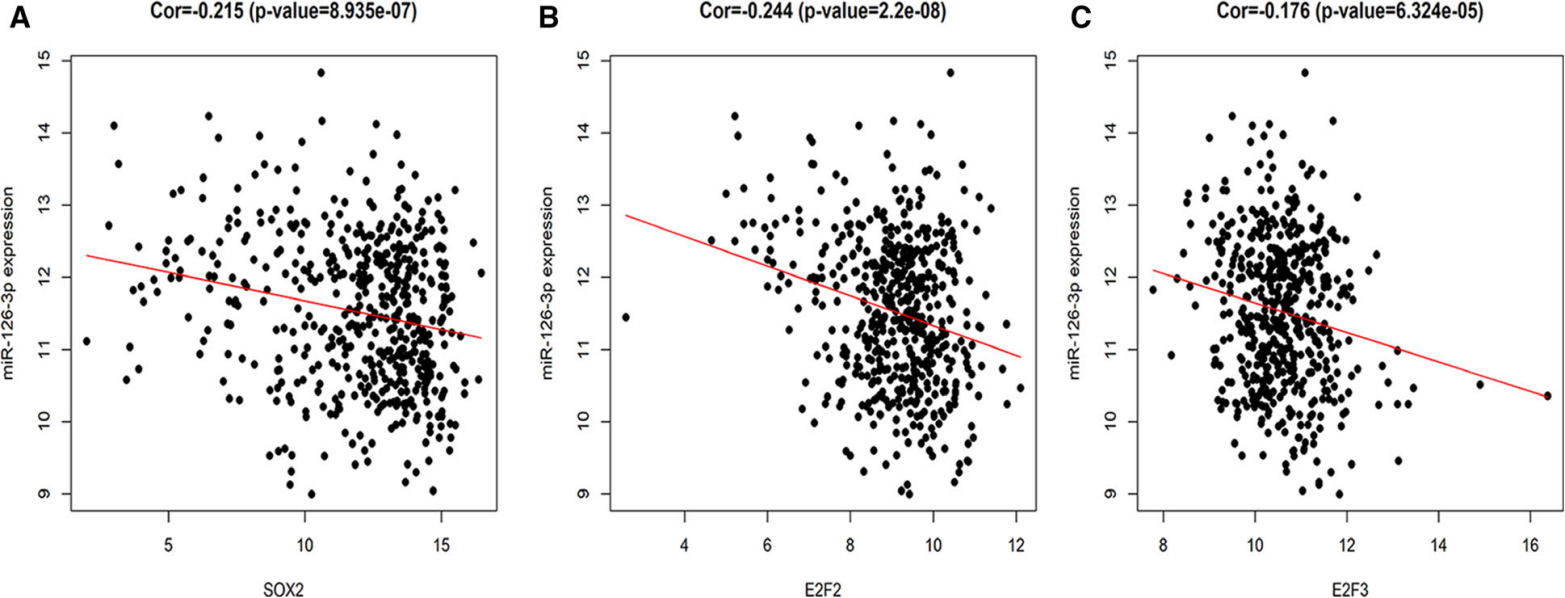
Pearson's correlation analysis of miRNA‐126‐3p expression and the expression of the three hub genes. (A) Correlation between miRNA‐126‐3p and *SOX2*. (B) Correlation between miRNA‐126‐3p and *E2F2*. (C) Correlation between miRNA‐126‐3p and *E2F3*. *P* < 0.05 indicated statistically significant.

Among the 42 target genes, *SOX2*, *E2F2*, and *E2F3* had more connections in the PPI network. We chose them as key genes to explore their clinical expressions in 502 tumor and 49 normal tissues from TCGA database. The expressions of *SOX2*, *E2F2*, and *E2F3* in LUSC and noncancerous cases are shown in Fig. 10A,C,E. The ROC curves evaluated the diagnostic ability of three hub genes (Fig. 10B,D,F). Figure 11 illustrates the correlation between three central genes and miRNA‐126‐3p. The three core gene expressions were significantly negatively correlated with the expression of miRNA‐126‐3p in LUSC.

## Discussion

Võsa *et al*. [26] discovered 15 abnormally expressed miRNAs in lung malignancies, including a low expression of miRNA‐126‐3p. This inspired the thought that these miRNAs might be potential biomarkers of lung cancer. To date, there have been many studies about miRNA‐126‐3p in the field of malignant lung tumors. However, the majority of studies about miRNA‐126‐3p in lung cancer are concentrated in non‐small‐cell lung cancer (NSCLC) or LUAD, and few reports in the literature concern the specific mechanism of action of miRNA‐126‐3p in LUSC. In this study, a GEO integration analysis, TCGA data mining, RT‐qPCR, and an integration analysis consistently indicated that in contrast to the noncancerous tissues, the miRNA‐126‐3p expression is significantly downregulated in LUSC tissues. We could distinguish LUSC from noncancerous tissues by the results of ROC curves derived from RT‐qPCR, all GSE datasets, TCGA data, and the integrated analysis. Based on previous findings [27], we confirmed that miRNA‐126‐3p was underexpressed in both LUAD and LUSC, and it played a role in tumor suppressor in LUAD and LUSC. The difference in the expression of miRNA‐126‐3p between T stages in the TCGA data suggests that the underexpression of miRNA‐126‐3p might stimulate the proliferation of LUSC tumor cells. Univariate and multivariate Cox analyses of TCGA data illustrate that miRNA‐126‐3p could be an independent prognostic factor in LUSC compared with other clinicopathological characteristics. A Kaplan–Meier survival curve analysis from TCGA data shows that the total survival time and median survival time of low‐expression miRNA‐126‐3p were greater than were those of high‐expression miRNA‐126‐3p. From this, we can infer that miRNA‐126‐3p has potential as a prognostic marker of LUSC. The results of this study are in concordance with those previously reported by Ulivi *et al*. [28], wherein the miRNA‐126‐3p expression in LUSC was shown to be related to OS, as well as its potential as an independent prognostic factor for LUSC.

To explore further the molecular mechanism of the miRNA‐126‐3p function in LUSC, we executed a functional analysis of bioinformatics on the predicted 42 target genes. Three functional analyses of GO showed that *SOX2*, *E2F2*, and *E2F3* were mainly enriched in GO–BP (regulation of transcription), GO–CC [transcription factor (TF) complex], and GO–MF (transcriptional activator and factor activity). Transcription regulation plays a pivotal role in orchestrating normal development and disease progression [29]. It had been proven that TFs can act as oncogenes or oncosuppressors [30]. Several investigations have confirmed that TFs and miRNA can regulate each other [31]. Therefore, we can infer that *SOX2*, *E2F2*, and *E2F3* act as TFs and can affect the biological process of LUSC through mutual regulation with miRNA126‐3p. In the KEGG pathway analysis, we noted that small cell lung cancer (SCLC), the cell cycle, pathways in cancer, prostate cancer, and microRNAs in cancer were all closely related to human cancer. Wang *et al*. [32] reported that miRNA‐126 and its target genes may be related to the lymph node metastasis of SCLC. Miko *et al*. [33] pointed out that miRNA‐126 can negatively regulate the proliferation of SCLC cells. Therefore, miRNA‐126 might be a pivotal biomarker shared by NSCLC and SCLC. Many studies have demonstrated the vital role of cell cycle apoptosis and cell cycle arrest in the biological processes of lung cancer [34, 35, 36]. We assume miRNA‐126‐3p may suppress the proliferation and infiltration of LUSC cells through cell cycle apoptosis and cell cycle arrest. However, this hypothesis requires further experimental validation. Interestingly, the two hub genes, *E2F2* and *E2F3*, were all involved in these cancer‐related pathways. Hence, we hypothesized that miRNA‐126‐3p regulated the biological function of LUSC by targeting *E2F2* and *E2F3*.

We selected three genes with the highest frequency as the hub genes from the PPI network: *SOX2*, *E2F2*, and *E2F3*. Because we used overexpressed genes in TCGA and GEO microarrays when we were predicting target genes, the expressions of these genes in tumor tissues were upregulated. Based on the miRNA‐126‐3p downregulated in tumor tissues, these overlapping and most frequently occurring genes can be used as target genes of miRNA‐126‐3p. The TF *SOX2* acted as a key regulator in biological processes, such as transcriptional regulation, cell proliferation, and oncogenesis [37], and the differential expression of *SOX2* has been confirmed in various cancers. de Vicente *et al*. [38] revealed that the *SOX2* expression was an important predictive biomarker of oral cancer risk in early stage patients, while Zhan *et al*. [39] identified that the aberrant expression of *SOX2* can inhibit esophageal squamous cell carcinoma development. Furthermore, an epigenomics analysis revealed that *SOX2* can drive tumor heterogeneity in LUSC [40]. Chang [41] found that the miRNA‐590‐5p/*SOX2* axis might be an underlying therapeutic direction in NSCLC. Although *SOX2* has been reported in NSCLC, there are few in‐depth studies on the mechanism of action between *SOX2* and MiRNA‐126‐3p in LUSC.

The E2F family plays an important role in cell cycle regulation, tissue homeostasis, and apoptosis [42, 43]. TFs *E2F2* and *E2F3* are two members of E2F family, and *E2F1‐3*, a subfamily of the E2F family, serves as a transcriptional activator [43]. Current studies indicate that *E2F2* and *E2F3* may act as vital regulators in several cancers. Lin *et al*. [44] confirmed that miRNA‐638 inhibited the biological behavior of breast cancer stem cells by interacting with *E2F2*. Moreover, it is well documented that* E2F2* can serve as an attractive therapeutic target and biomarker for ovarian cancer [42]. *E2F2* worked as an activator in the tumor invasion of NSCLC, and it was implicated in the proliferation and poor prognosis of NSCLC cells [45]. The specific mechanism of action between related miRNA and *E2F3* has been reported in the literature on hepatocellular carcinoma [46], bladder cancer [47], ovarian cancer [48], LUAD [49], and gastric cancer [50]. The mechanisms of interaction of *E2F2‐3* and miRNA‐126‐3p in LUSC have not yet been reported. We speculate that miRNA‐126‐3p may target *E2F2* and *E2F3* to restrain the cell proliferation and cancer progression of LUSC. We consider that miRNA‐126‐3p regulates the elevated expressions of core genes, *SOX2*, *E2F2*, and *E2F3*, and it may help elucidate the carcinostatic functions of miRNA‐126‐3p in LUSC. This must also be confirmed by further experimental function studies.

This is the first in‐depth study on and discussion of the mechanism of miRNA‐126‐3p in LUSC. By combining RT‐qPCR, bioinformatics, and an integrated analysis, we confirmed that the downregulation of miRNA‐126‐3p in LUSC was involved in several biological processes that promote the occurrence, progression, and poor prognosis of LUSC. The key role of miRNA‐126‐3p in LUSC has provided new ideas and directions for the diagnosis of and molecular therapy for LUSC in the future.

The study has several shortcomings. First, it is a single‐center study, so the number of clinical samples used for the RT‐qPCR analysis is small, and more clinical tissue samples are needed for more accurate and persuasive results. Second, it lacks *in vitro* studies to validate the effect of miRNA‐126‐3p knockdown or overexpression in LUSC. In future studies, it will be necessary to add well‐designed *in vitro* experiments to validate the results and hypotheses of this study. Third, to investigate the early diagnostic value of miRNA‐126‐3p for LUSC, we attempted to screen the high throughput data from the GEO, ArrayExpress, SRA, and Oncomine datasets. Unfortunately, no data on the miRNA‐126‐3p expression in LUSC patients were available from blood, serum, or plasma samples. Five microarrays (GSE17681, GSE24709, GSE40738, GSE61741, and GSE64591) were obtained from blood in NSCLC patients, which could reflect the potential diagnostic value for the whole NSCLC population. Indeed, the expression of miRNA‐126‐3p in NSCLC patients was significantly lower than in healthy controls (data not shown). However, the clinical implication of miRNA‐126‐3p in the early diagnosis of LUSC patients must be confirmed in future.

## Conclusion

In this study, a comprehensive analysis of RT‐qPCR, TCGA, and GEO datasets illustrated that miRNA‐126‐3p is underexpressed in LUSC tissues. A low expression of miRNA‐126‐3p suppressed the development of LUSC, whereas a high expression of miRNA‐126‐3p was associated with a better LUSC prognosis. MiRNA‐126‐3p may act as a tumor inhibitor in LUSC, and it suppresses the proliferation of LUSC cells. In summary, miRNA‐126‐3p may be a promising biomarker for LUSC diagnosis and prognosis.

## Conflict of interest

The authors declare no conflict of interest.

## Author contributions

All authors have contributed to this study for submission. S‐WC performed the data extraction and statistical analysis and drafted the paper. S‐WC and H‐PL conceived and designed the study and edited the manuscript. GC, JY, H‐FZ, and W‐JM contributed to the design of the study, supervised all experiments, and corrected the paper. W‐YH, X‐MW, S‐PH, LG, JLi, Z‐WF, PC, and G‐QZ collected, extracted, and analyzed the data. X‐JL, Z‐GH, Z‐YL, JLu, D‐PY, and T‐QG critically revised the manuscript. All authors have contributed to, read, and approved the final manuscript for submission.

## Supporting information


**Fig. S1.** Stability of 13 potential references in lung squamous cell carcinoma (LUSC) tissues and cells evaluated by geNorm and NormFinder. A total of 12 cases of LUSC and their corresponding adjacent non‐tumor lung tissues was used to test the references (a: geNorm, c: NormFinder). Five different siRNAs were transfected into CALU1 cells as well (b: geNorm, d: NormFinder).
**Table S1.** Stability values of potential internal references based on geNorm and NormFinder.

## Data Availability

The data of TCGA were obtained from the website of TCGA database (https://tcga‐data.nci.nih.gov/docs/publications/tcga/). The data of GSE datasets were obtained from the website of the GEO database (https://www.ncbi.nlm.nih.gov/geo/). The data of the First Affiliated Hospital of Guangxi Medical University cohort used to support the findings of this study are available from the corresponding author upon request.
