# Supplementary material for: Downregulation of miRNA‐126‐3p is associated with progression of and poor prognosis for lung squamous cell carcinoma
Source: FEBS Open Bio. 2020 Jul 14;10(8):1624–41. doi: 10.1002/2211-5463.12920 (PMC7396450; doi:10.1002/2211-5463.12920)
Supplement: Supplementary file 1 — Fig. S1. Stability of 13 potential references in lung squamous cell carcinoma (LUSC) tissues and cells evaluated by geNorm and NormFinder. A total of 12 cases of LUSC and their corresponding adjacent non‐tumor lung tissues was used to test the references (a: geNorm, c: NormFinder). Five different siRNAs were transfected into CALU1 cells as well (b: geNorm, d: NormFinder). Table S1. Stability values of potential internal references based on geNorm and NormFinder. [file FEB4-10-1624-s001.docx]

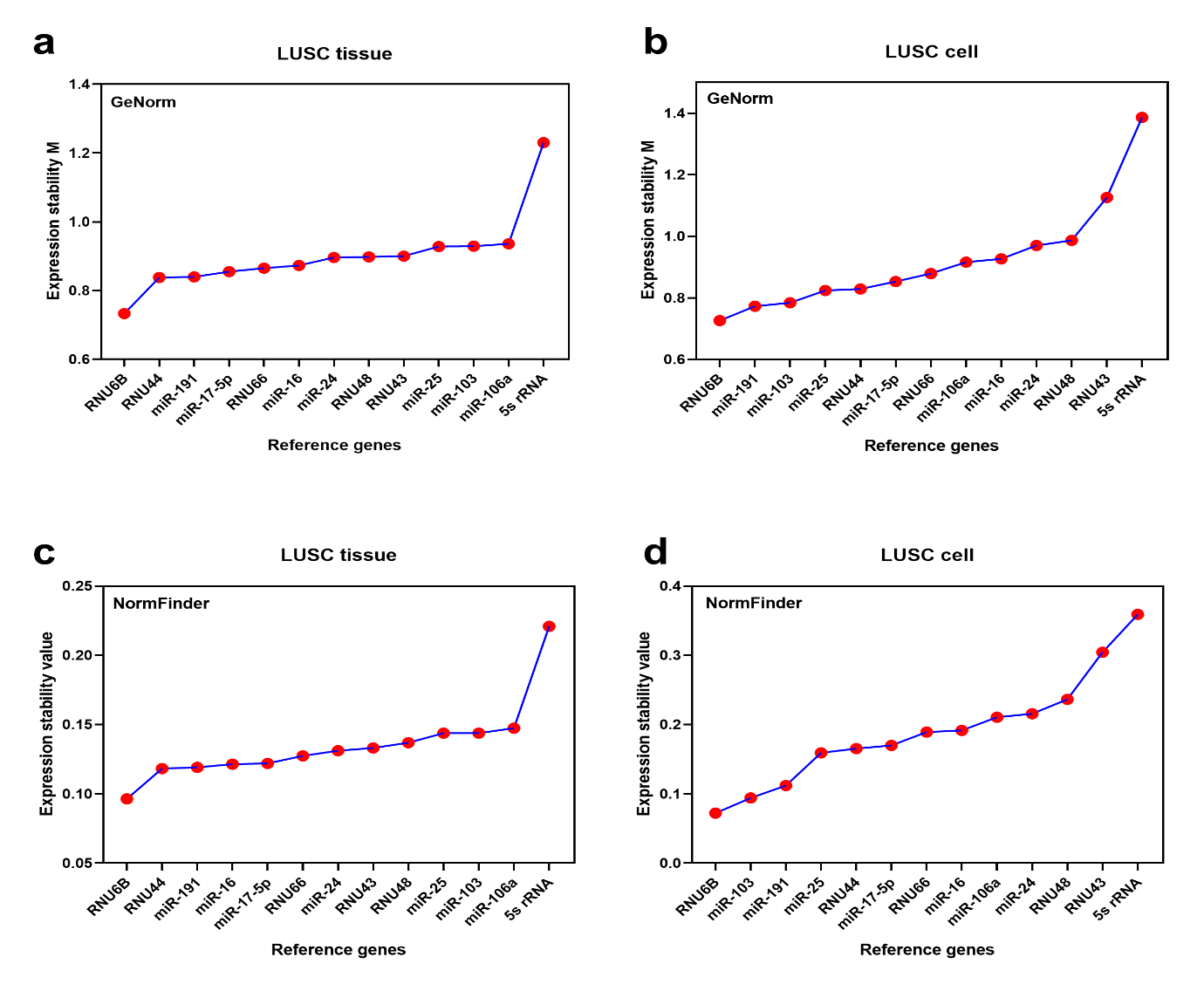


**Supplementary Figure 1**. Stability of 13 potential references in lung squamous cell carcinoma (LUSC) tissues and cells evaluated by geNorm and NormFinder. A total of 12 cases of LUSC and their corresponding adjacent non-tumor lung tissues was used to test the references (**a**: geNorm, **c**: NormFinder). Five different siRNAs were transfected into CALU1 cells as well (**b**: geNorm, **d**: NormFinder).

**Supplementary table 1**. Stability values of potential internal references based on geNorm and NormFinder.

| Reference genes | GeNorm | |  | NormFinder | |
| --- | --- | --- | --- | --- | --- |
|  | LUSC tissue | LUSC cell |  | LUSC tissue | LUSC cell |
| RNU6B | **0.733** | **0.726** |  | **0.096** | **0.072** |
| RNU48 | 0.898 | 0.987 |  | 0.137 | 0.236 |
| RNU66 | 0.865 | 0.879 |  | 0.127 | 0.189 |
| RNU44 | 0.838 | 0.829 |  | 0.118 | 0.165 |
| RNU43 | 0.900 | 1.126 |  | 0.133 | 0.304 |
| 5s rRNA | 1.230 | 1.386 |  | 0.221 | 0.359 |
| miR-16 | 0.873 | 0.927 |  | 0.121 | 0.191 |
| miR-17-5p | 0.855 | 0.853 |  | 0.122 | 0.170 |
| miR-24 | 0.896 | 0.970 |  | 0.131 | 0.216 |
| miR-25 | 0.928 | 0.824 |  | 0.144 | 0.159 |
| miR-103 | 0.929 | 0.784 |  | 0.144 | 0.094 |
| miR-106a | 0.936 | 0.916 |  | 0.147 | 0.211 |
| miR-191 | 0.840 | 0.773 |  | 0.119 | 0.112 |

LUSC, lung squamous cell carcinoma.
